# Elucidation of major contributors involved in nitrogen removal and transcription level of nitrogen-cycling genes in activated sludge from WWTPs

**DOI:** 10.1038/srep44728

**Published:** 2017-03-15

**Authors:** You Che, Peixin Liang, Ting Gong, Xiangyu Cao, Ying Zhao, Chao Yang, Cunjiang Song

**Affiliations:** 1 Key Laboratory of Molecular Microbiology and Technology for Ministry of Education, Nankai University, Tianjin, 300071, China; 2 School of Life Science, Liaoning University, Shenyang, 110036, China; 3 Department of Biological Science, Jining Medical University, Jining, 272067, China; 4 Department of Microbiology, College of Life Sciences, Nankai University, Tianjin, 300071, China

## Abstract

We investigated nitrogen-cycle bacterial communities in activated sludge from 8 municipal wastewater treatment plants (WWTPs). Redundancy analyses (RDA) showed that temperature was the most significant driving force in shaping microbial community structure, followed by influent NH_4_^+^ and total nitrogen (TN). The diversity of ammonia oxidizing and nitrite reducing bacteria were investigated by the construction of *amoA, nirS* and *nirK* gene clone libraries. Phylogenetic analysis indicated that *Thauera* and *Mesorhizobium* were the predominant nitrite reducing bacteria, and *Nitrosomonas* was the only detected ammonia oxidizing bacteria in all samples. Quantification of transcription level of *nirS* and *nirK* genes indicated that *nirS*-type nitrite reducing bacteria played the dominant roles in nitrite reduction process. Transcription level of *nirS* gene positively correlated with influent NH_4_^+^ and TN significantly, whereas inversely linked with hydraulic retention time. Temperature had a strong positive correlation to transcription level of *amoA* gene. Overall, this study deepened our understanding of the major types of ammonia oxidizing and nitrite reducing bacteria in activated sludge of municipal WWTPs. The relationship between transcription level of nitrogen-cycle genes and operational or environmental variables of WWTPs revealed in this work could provide guidance for optimization of operating parameters and improving the performance of nitrogen removal.

Highly complex activated sludge systems have been the most commonly biological process in wastewater treatment plants (WWTPs), where a wide variety of bacteria play dominant roles in pollutant degradation and removal^1^, being thus an ideal model for microbial ecology study^2^. Our knowledge of microbial community composition and diversity in various municipal and industrial WWTPs has been advanced^3^,^4^, mainly because of the aid of high-throughput sequencing (HTS) technique. Additionally, studies have been carried out to identify the main environmental and operational factors influencing the microbial community composition^5^,^6^. However, knowledge regarding the major driving force behind the community structure of full-scale municipal WWTPs is incomplete. Despite rapidly increasing understanding of bacterial community structure, research targeting the diversity of functional bacteria associated with nitrogen removal in activated sludge needs more attention, which expected to influence the performance of WWTPs.

Total nitrogen (TN) removal from wastewater is one of the major tasks in biological WWTPs, which is driven by the combination of nitrification and denitrification. Ammonia is oxidized to nitrite by ammonia monooxygenase of ammonia oxidizing bacteria (AOB), which is believed to be the key step in nitrification process. Therefore, *amoA* gene, which encoding subunits of this enzyme was widely used as biomarker to investigate the AOB in different environments, such as in intermittent aeration sequencing batch reactors^7^ and aerated full-scale activated sludge bioreactor^8^. Copper or cytochrome cd1-containing nitrite reductase is the key enzyme in the denitrification process catalyzes the nitrite into nitric oxide (NO), subunits of which was encoded by *nirK* or *nirS* gene, respectively. Community composition of nitrite reducing bacteria based on *nirK* and *nirS* genes was investigated by a number of studies in different environments, such as coastal aquifers^9^ and marine sediments^10^.

Although previous studies revealed the nitrogen-cycle microbial communities in industrial WWTPs^11^, comprehensive research aiming at the community structure of ammonia oxidizing bacteria and nitrite reducing bacteria in multiple activated sludge of municipal WWTPs was limited. It has long been accepted that the diversity of functional bacteria closely correlates with the removal efficiency of pollutants, it also influences the stability and sustainability of WWTPs. So, elucidating the influencing factors behind the biodiversity of nitrogen-cycle bacteria is particularly important to optimize the condition for nitrogen removal.

Exploring the abundance of *amoA, nirS* and *nirK* genes based on DNA level may introduce a bias because it could not reflect the active bacteria. In contrast, research concerning transcription level of *amoA, nirS* and *nirK* genes can make us understand the activity of ammonia oxidizing and nitrite reducing bacteria in activated sludge^12^,^13^, which were also conducted in our study. In particular, exploring the influence of specific deterministic operational and environmental factors on transcription level of these genes in activated sludge could provide valuable information for improving the performance of nitrogen removal in WWTPs.

Overall, the primary purposes of this study were: (i) to explore microbial community composition in activated sludge from multiple WWTPs along geographical gradients and driving factors in shaping the community structure; (ii) to determine core predominant functional microorganisms involved in nitrogen removal; (iii) to preliminarily discuss the relationship between the transcription level of *amoA, nirS* and *nirK* genes and operational and environmental variables.

## Results

### Diversity of microbial communities

For the bacterial community analyses in the 8 activated sludge samples, 62,806 effective reads were obtained after filtering the low quality reads and trimming the barcodes, adapters and primers. The numbers of OTUs, Good’s coverage, Chao 1 and Shannon parameters at 3% cutoff level are summarized in Supplementary Table S1. The highest richness was found in the DL sample from Tianjin based on OTU numbers, followed closely by those from Liaoning and Shandong, whereas samples collected from Fujian displayed considerable lower richness, which were confirmed by the rarefaction curves (Supplementary Fig. S1). The α-diversity of various samples, which is indicative of Shannon index, displayed the same trend as the number of OTUs. Principal coordinates analysis (PCoA) was conducted to evaluate similarities among samples. As shown in Supplementary Fig. S2, those geographically closer samples except for FJ1 and FJ2 were clustered together in PCoA.

### Analysis of bacterial community structure at the phylum level

The effective bacterial sequences in each sample were assigned to different taxonomic levels using the RDP classifier at 80% threshold. The relative abundance of the bacterial community at the phylum level for each sample is shown in Supplementary Fig. S3. *Proteobacteria* was the dominant phylum in all samples, accounting for 27–52.5% of total effective sequences, except for the samples RZ and FJ2, in which the most abundance phylum was *Bacteroidetes* (42.6% and 43.8%, respectively). *Chlorobi* and *Acidobacter* were the subdominant groups in all samples, comprising 2.7–8.1%, 2.8–5.1% of total effective sequences, respectively, except for the sample FJ1. *Chloroflexi* and *Firmicutes* were detected in all samples but presented particularly high abundance in FJ1, accounting for 25.9% and 22.4%, respectively. Notably, *Synergistetes* was the only major phylum (9.0%) in FJ1 sample.

### Analysis of bacterial community structure at the genus level

**Figure 1:**
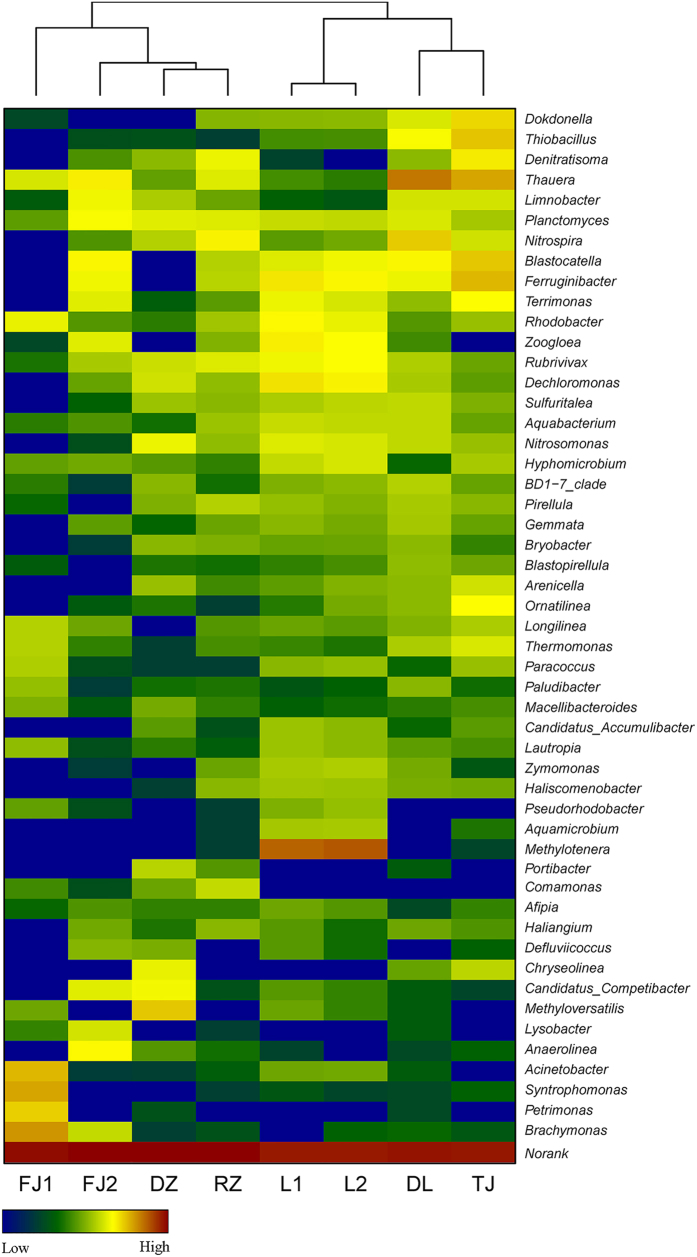
Heatmap of the most abundant bacterial genera in 8 activated sludge samples, cluster analysis was performed on the 51 most represented genera (abundance >0.1%) based on Bray-Curtis similarity index.

The top 51 abundant genera which abundance were higher than 0.1% of the total classified sequences were selected, their profiles among different samples were shown in Fig. 1. Comparative analysis revealed a core set of bacteria were shared across the 8 activated sludge samples, among the 51 assigned genera, 14 genera were shared by all the samples, and 30 genera were shared by at least 7 samples, accounting for 85% of all the classified sequences (see Supplementary Table S2). It is reasonable to believe that these widely distributed core genera play key roles in wastewater treatment system^14^. However, several genera showed some geographical characteristics. For example, *Methylotenera* was more abundant in L1 and L2 samples, and 4 genera (i.e., Acinetobacter, *Syntrophomonas, Petrimonas*, and *Brachymonas*) were more abundant in FJ1 sample. Cluster analysis revealed that bacterial community structures at the genus level formed a relatively close cluster separated along the geographical locations.

### Phylogenetic analysis of ammonia oxidizing and nitrite reducing bacteria

Considering the key roles of nitrogen-cycle related ammonia oxidizing and nitrite reducing bacteria in maintaining the treatment performance of activated sludge, clone libraries were constructed using *amoA, nirS*, and *nirK* genes as markers to investigate the distribution and diversity of the functional bacterial community in different activated sludge samples.

**Figure 2:**
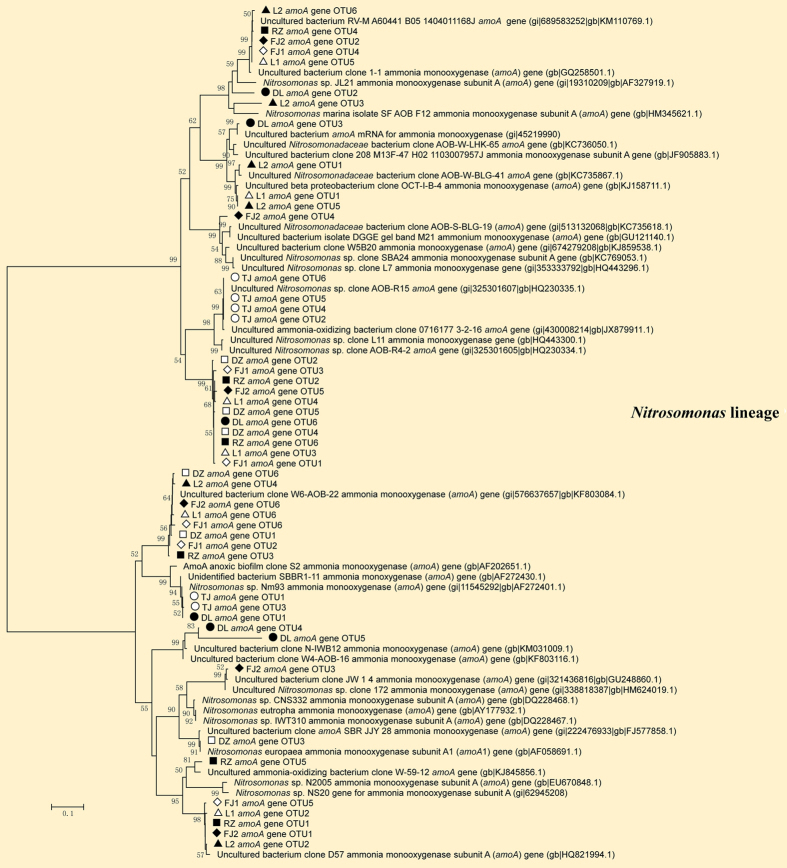
Phylogenetic tree constructed by the maximum likelihood method based on bacterial *amoA* gene sequences from 8 activated sludge samples and bootstrap analysis is carried out with 1,000 replicates. Only bootstrap values greater than 50% are indicated on tree. The scale bar represent 10% nucleotide divergence.

**Figure 3:**
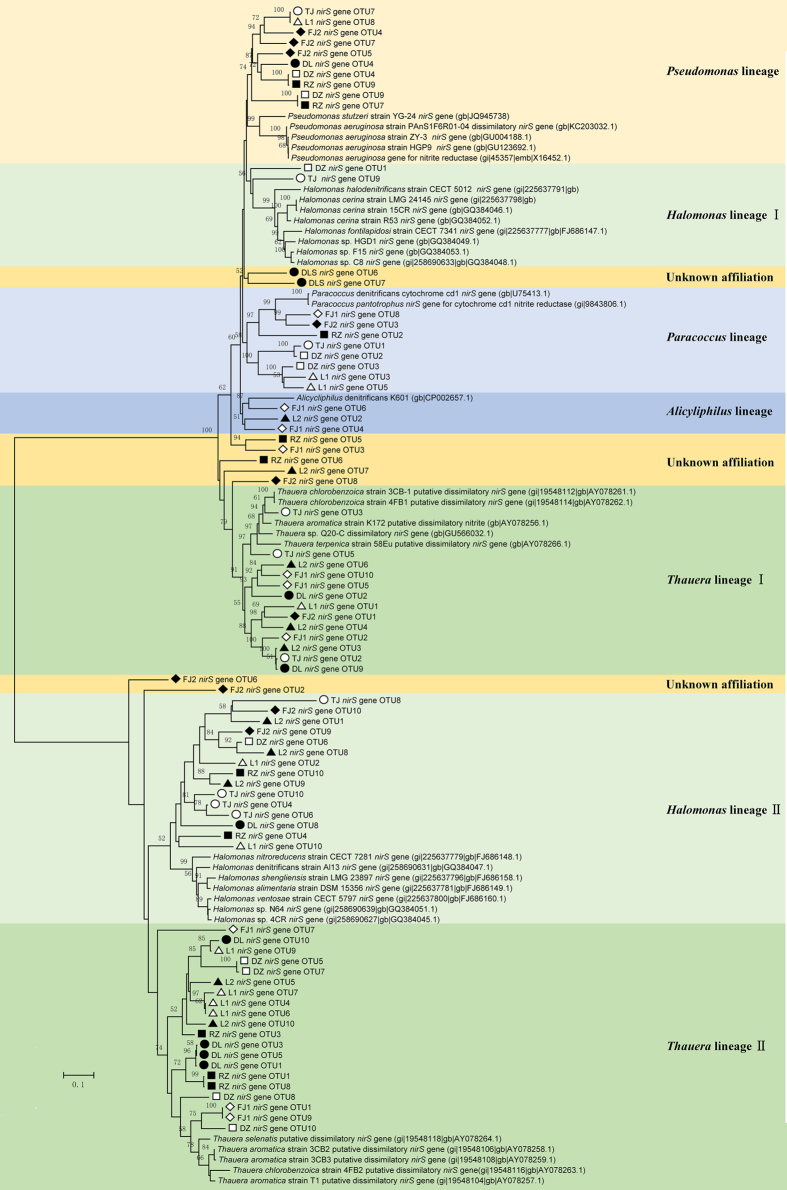
Phylogenetic tree constructed by the maximum likelihood method based on bacterial *nirS* gene sequences from 8 activated sludge samples and bootstrap analysis is carried out with 1,000 replicates. Only bootstrap values greater than 50% are indicated on tree. The scale bar represent 10% nucleotide divergence.

**Figure 4:**
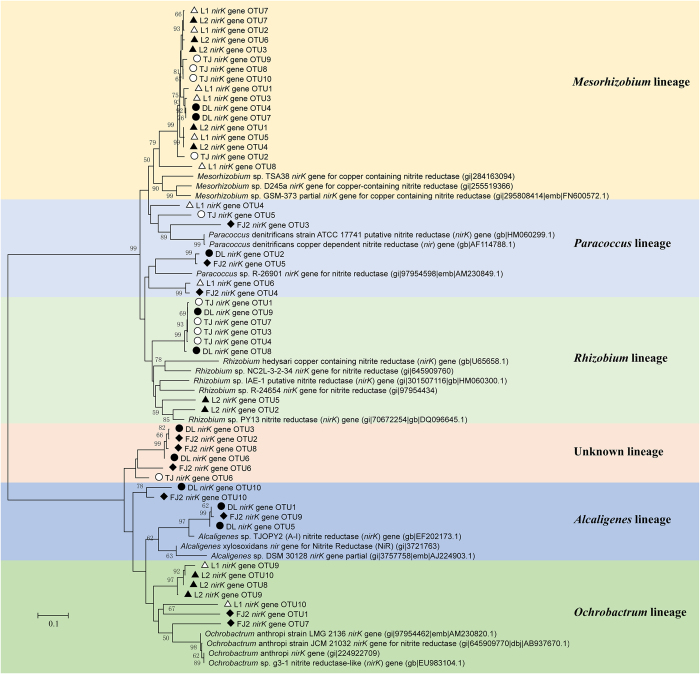
Phylogenetic tree constructed by the maximum likelihood method based on bacterial *nirK* gene sequences from 5 activated sludge samples and bootstrap analysis is carried out with 1,000 replicates. Only bootstrap values greater than 50% are indicated on tree. The scale bar represent 10% nucleotide divergence.

Phylogenetic analysis revealed that bacterial *amoA* sequences in all samples were affiliated to the *Nitrosomonas* lineage (Fig. 2), whereas no sequences belonging to the *Nitrosospira* lineage were observed, which are consistent with the results of high-throughput sequencing in which no *Nitrosospira* sequences were found either. Nitrite reduction is the key step in the denitrification reaction catalyzed by two functionally equivalent but structurally different forms of nitrite reductases, cytochrome cd1 and copper containing nitrite reductases encoded by the genes of *nirS* and *nirK*, respectively. All the *nirS* sequences obtained in this study fell into 10 distinct lineages belonging to 5 different genera, including *Thauera, Halomonas, Paracoccus, Pseudomonas*, and *Alicyliphilus, Thauera* was shared by all the samples (Fig. 3). As for *nirK*, clone libraries were successfully constructed only in 5 samples (L1, L2, TJ, DL and FJ2). The phylogenetic tree based on *nirK* gene grouped into 6 major clusters, which were affiliated with the genera *Mesorhizobium, Paracoccus, Rhizobium, Alcaligenes, Ochrobactrum*, and unknown lineage (Fig. 4). *Mesorhizobium* was detected in all the samples except FJ2, and *Paracoccus* was not observed in L2.

**Figure 5:**
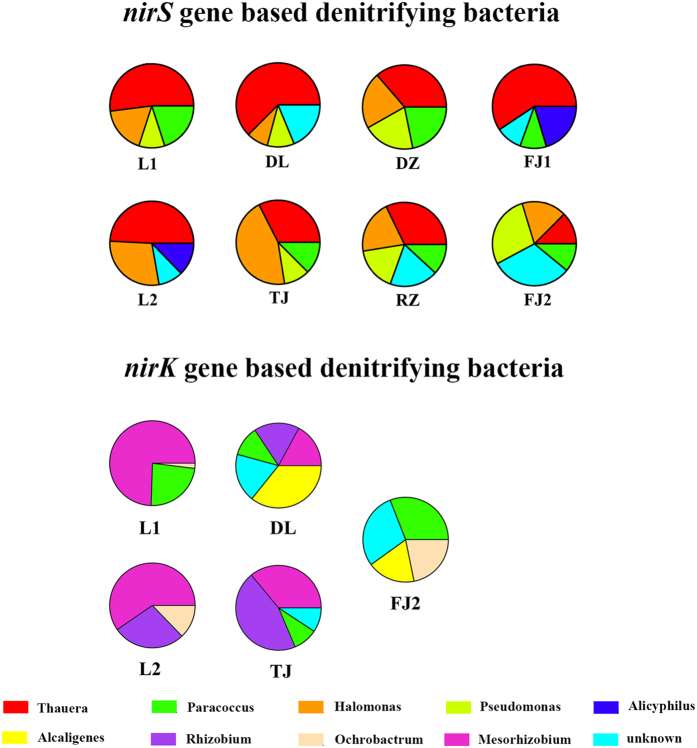
Pie chart showing the distribution pattern of denitrifying bacteria, each pie chart represents the relative abundance of different denitrifying bacteria in the activated sludge samples indicated above.

The relative abundance of the nitrite reducing bacteria of each sample was detected (Fig. 5). As a result, *Thauera* and *Mesorhizobium* dominated the nitrite reducing bacterial community, accounting for 41.5% and 43.8% of all the sequences, respectively, suggesting that some core bacteria play the key functional roles in nitrite reduction process.

### Quantification of the ammonia oxidizing and nitrite reducing bacterial community abundance

**Figure 6:**
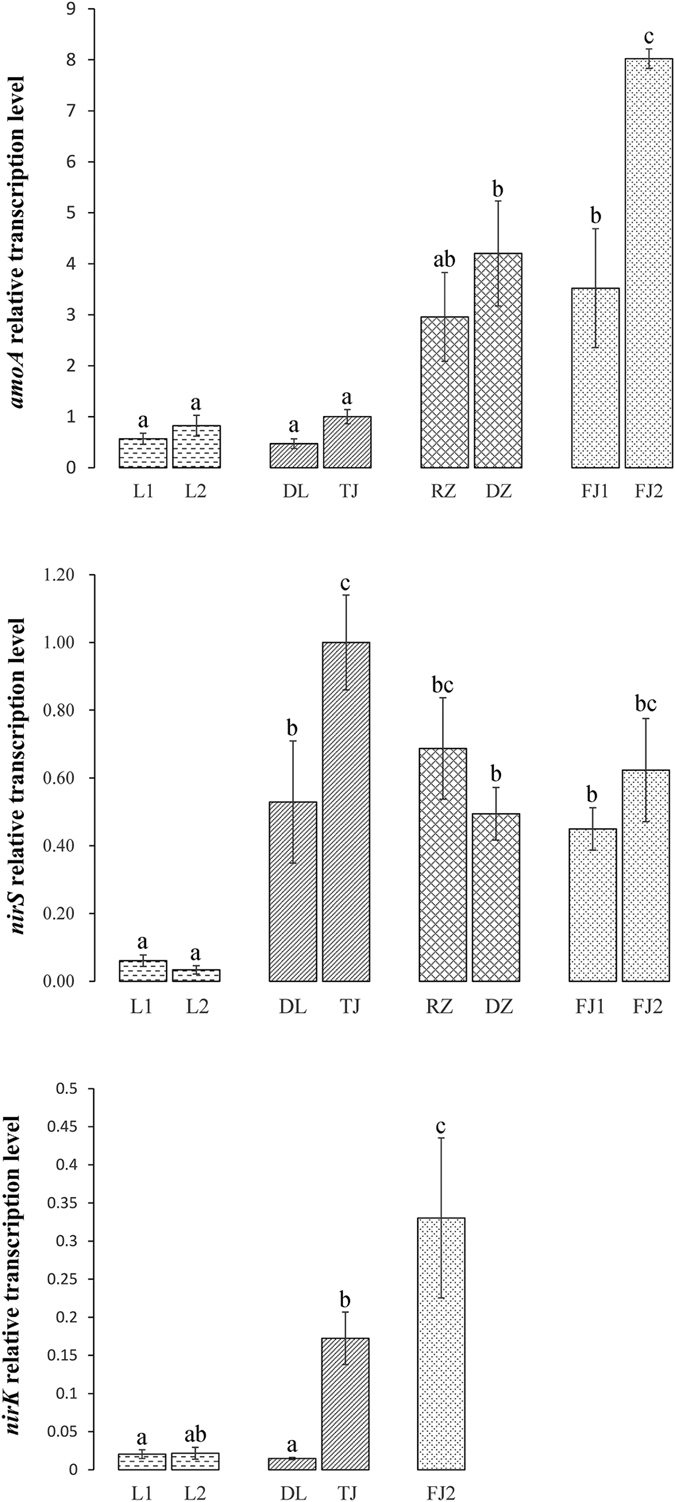
Relative transcription level of *amoA, nirS, nirK* genes in the 8 activated sludge samples revealed by qRT-PCR. Significant (*p* < 0.05) differences as calculated by one-way ANOVA were indicated by different letters. The transcription level values were expressed as the fold change among these different samples.

Evaluation of the ammonia oxidizing and nitrite reducing bacteria abundance by quantitative real-time PCR (qRT-PCR) based on transcription level could significantly enhance our understanding of active groups in activated sludge. In this study, transcription level of the *amoA, nirS* and *nirK* genes normalized to total bacterial 16S rRNA was analyzed by qRT-PCR (Fig. 6). The relative abundance of *amoA* gene varied considerably among the samples from different locations. Relative transcription levels of *amoA* were approximately 3- to 8-fold higher in Shandong and Fujian samples compared to Liaoning and Tianjin samples. The similar *nirS* abundances were detected in all the samples except Liaoning in spite of the different locations. In contrast, 2 samples from Liaoning showed a significant lower *nirS* transcription level than other samples. Transcription level of *nirK* gene differed substantially among the 5 samples. Meanwhile, distinct differences of transcription level could also be detected among those geographically closer samples, such as *amoA* gene in FJ1 and FJ2 and *nirK* gene in DL and TJ. Notably, transcription level of the two functional genes (*nirS* and *nirK*) showed significant differences in all the samples. Our results found a predominance of *nirS* transcription level over *nirK* in all the samples, suggesting that *nirS*-type nitrite reducing bacteria may be the main contributor to nitrite reduction in activated sludge from municipal WWTPs.

## Discussions

**Figure 7:**
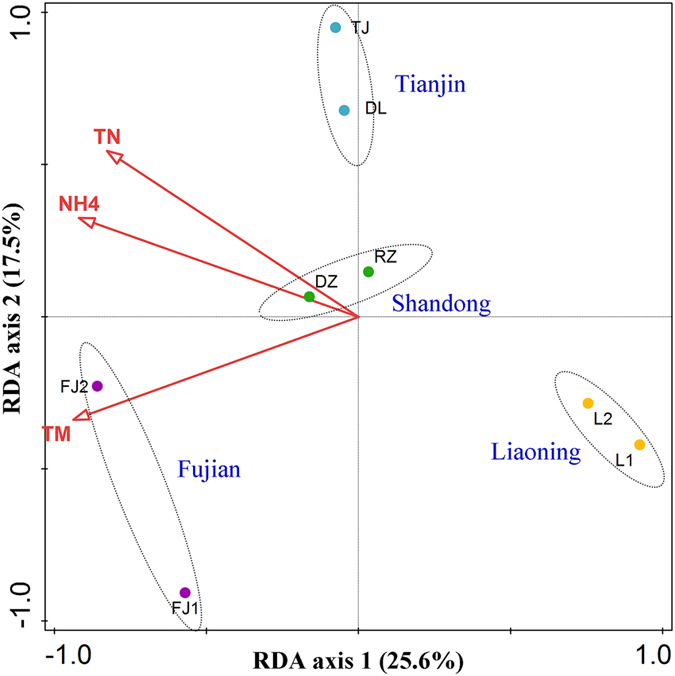
Redundancy analysis (RDA) of high throughput data and variables in the 8 samples. Only the explanatory variables significantly (*p* < 0.05) explaining microbial community variations are presented. Arrows indicate the direction and strength of variables correlated with bacterial community structure.

It has been accepted that bacterial communities intimately relate to ecological functions and influence the removal of nutrients and pollutants. The bacteria found in our study were frequently detected in previous studies and have been considered the typical activated sludge bacterial communities^15^. Investigating the correlation of bacterial community with surrounding environment and operational parameters could promote ecosystem sustainability and stability. A gradient ordination method (RDA) was conducted to discern the major factors shaping activated sludge bacterial community in Fig. 7, in which the length of the arrow indicates the strength of the correlation of the factor with bacterial community composition. According to the permutation test, 3 major factors including temperature, influent NH_4_^+^ and TN imposed significant effects (*p* < 0.05) on the bacterial community, in which temperature was a critical factor in driving the community variation. Other observed variables, including influent COD, pH, dissolved oxygen (DO), and hydraulic retention time (HRT) may explain, to some extent, the microbial community structure but had no statistically significant (*p* > 0.05).

Although bacterial community structures at the genus level formed a close cluster separated along the geographical locations (Fig. 1), core genera populations existed in all the samples. Among the shared genera, members of *Thauera, Rhodobacter, Hyphomicrobium* and *Paracoccus* are functionally important denitrifiers as previously reported^16^. *Planctomyces, Prirellula*, and *Gemmata*, which belong to the *Planctomycetes* phylum, are considered major players in the global nitrogen and carbon cycles. Our results suggested that environment and operational parameters exerted no considerable impacts on the existence of core bacterial community, the same operational mode (A/O) maybe a key factor in shaping the core genera populations since previous study found that the reactor configuration was the only parameter influencing community diversity^5^.

In our case, phylogenetic studies based on the construction of clone libraries of 3 functional genes (*amoA, nirS* and *nirK*) identified high proportion of core set of nitrogen cycle genera shared by all the samples, such as *Nitrosomonas* for ammonia oxidization and *Thauera* or *Mesorhizobium* for nitrite reduction. Clone libraries based on *nirK* gene were constructed only in 5 samples, which are similar with the previous results that *nirS* sequences could be amplified whereas *nirK* sequences were only detected in certain samples in pacific northwest marine sediment communities^10^. Diversity of nitrite reducing community distribution varied slightly based on *nirS* and *nirK* genes, although previous studies found that *nirK*-type nitrite reducing bacteria showed greater variation than *nirS*-type in the black sea suboxic zone^17^, and that *nirS* based community showed higher diversity in full^18^ or laboratory scale^19^ wastewater treatment systems.

Remarkably, *Nitrosomonas* was the only detected functional community involved in the ammonia oxidizing process. Indeed, previous reports have demonstrated that temperature was a key factor influencing the lineages of ammonia oxidizing bacteria (AOB) in different ecological systems, such as in soil^20^. DO was also recognized as a critical parameter influencing the AOB activity^21^. In this study, the growth rate of *Nitrosomonas* out-compete other AOB lineages within the temperature ranging from 14.2 °C to 30 °C and the DO ranging from 1.7 mg l^−1^ to 2.8 mg l^−1^, allowing for the predominant existence of *Nitrosomonas* in all samples.

Based on the predominance of transcription level of *nirS* over *nirK* throughout all the samples, we demonstrated that *nirS*-type nitrite reducing bacteria dominated in the nitrite reducing process. The findings based on RNA level are straightforward since expression of *nirS* or *nirK* is necessarily associated with the mRNA of active bacteria. In our case, *nirS* transcription level was orders of magnitude higher than *nirK*. What’s more, *nirK* gene was amplified only in 5 samples, this phenomenon may be duo to that *nirK*-type nitrite reducing bacteria are more sensitive than *nirS*-type to environmental factors^22^. So, we assumed that nitrite reductase encoded by *nirS* gene may have higher substrate affinity than that encoded by *nirK* gene, which endowed competitive advantages for *nirS*-type nitrite reducing bacteria. Also, a mutually exclusive phenomenon between the two functional equivalent genes is expected. Since coexisting nitrite reductase genes in a denitrifier genome has not been found to date, and elimination and replacement could occur between the two types of *nir* genes in denitrifier^23^. Coexistence of both *nir* genes may implies functional redundancy, which means that loss of one type is allowed to maintain the stability of ecosystem function^24^.

Different transcription levels of functional genes related to nitrogen metabolic pathway have been observed in the samples. Furthermore, elucidating correlations of transcription level with environmental and operational parameters could improve our understanding of the conditions that favor the expression of functional genes. Pearson correlation analysis was conducted (Supplementary Fig. S4). The results showed that transcription level of *amoA* gene was positively correlated with temperature (*r* = 0.807; *p* = 0.015), whereas other variables including NH_4_^+^ (*r* = 0.681; *p* = 0.063), TN (*r* = 0.418; *p* = 0.303) and HRT (*r* = −0.250; *p* = 0.550) exerted no significant influence on the transcription level of *amoA* gene. For *nirS* and *nirK* genes, TN was positively correlated with the corresponding *nirS* gene transcription level most significantly (*r* = 0.918; *p* = 0.001), followed by correlation with HRT negatively (*r* = −0.877; *p* = 0.004) and with NH_4_^+^ positively (*r* = 0.747; *p* = 0.033). Temperature had a weak influence on the transcription level of *nirS* and *nirK* genes although the significance is not obvious. However, other variables monitored in our study including COD, pH and DO showed no correlation with transcription level of *amoA, nirS* and *nirK* genes (data not shown).

## Conclusion

In this study, high throughput sequencing of the bacterial 16 S rRNA gene combined with clone libraries (*amoA, nirS* and *nirK*) were applied to investigate nitrogen-cycling bacterial communities in activated sludge. Community structure analysis revealed that a core set of bacteria existed in all samples. Phylogenetic analysis of ammonia oxidizing and nitrite reducing bacteria showed that *Nitrosomonas, Thauera* and *Mesorhizobium* were the most dominant functional communities involved in nitrogen removal. The transcriptional level of *nirS* gene was much higher than *nirK* gene, which revealed the major contributor in the nitrite reduction process. Meanwhile, we preliminary discussed the factors affecting the transcriptional level of the nitrogen removal genes, which provided more information to optimize the nitrogen removal efficiency.

## Methods

### Sampling of activated sludge from WWTP

Activated sludge samples were collected from aeration tanks of 8 full-scale municipal WWTPs in different cities of China during October. The 8 WWTPs exhibited stable operation performance. The operation parameters and wastewater characteristics of these WWTPs are summarized in Supplementary Table S3. The activated sludge samples, which were supplemented by LifeGuard Soil Preservation Solution (MO-BIO Laboratories, Inc., CA), were transported on ice to our laboratory and stored at −80 °C prior to DNA and RNA extraction.

### Nucleic acid extraction and cDNA synthesis

DNA and RNA of the activated sludge samples were extracted with the SoilGen DNA Kit (CWBIO, Beijing, China) and RNA PowerSoil Total RNA Isolation Kit (MO-BIO Laboratories, Inc., CA, USA), respectively, according to the manufacturers’ instructions. The concentration and quality of the extracted DNA and RNA were determined with a microspectrophotometry (Nanodrop ND-2000, NanoDrop Technologies, Wilmington, DE, USA). DNA solution was stored at −20 °C for further analysis. The cDNA was synthesized from RNA using the Prime Scrip RT Master Mix (TaKaRa, Dalian, China) according to the manufacturer’s instructions after the treatment with DNase I at 37 °C for 30 min. The 10 μl reaction system contained 2 μl 5X PrimeScript RT Master Mix and 500 ng total RNA, and the reverse transcription program was as follows: 37 °C, 15 min, 85 °C, 5 sec. Then the cDNA was stored at −80 °C for further qRT-PCR analysis.

### PCR amplification, cloning, sequencing and phylogenetic analysis

Three key nitrogen-cycle genes (*amoA, nirK* and *nirS*) were amplified from activated sludge metagenomic DNA using the primers described in Supplementary Table S4. The 50-μl PCR mixture was composed of 0.2 μM of each primer, 25 μl of NI-Taq PCR Master Mix (NEWBIO INDUSTRY, China), and 20–50 ng of template DNA. The PCR protocols were set as below: an initial denaturation at 95 °C for 10 min, followed by 30 cycles of 94 °C for 30 s, annealing at 53 °C (for *amoA*) or 56 °C (for *nirS* and *nirK*) for 30 s, and elongation at 72 °C for 60 s, with a final extension at 72 °C for 10 min.

Prior to cloning, the PCR products were purified using AxyPrep DNA Gel Extraction Kit (AXYGEN, Hangzhou, China). Subsequently, the purified PCR products were ligated to Ptz57R/T vector (Thermo, Waltham, MA, USA) according to the manufacturer’s instructions and transformed into *E. coli* DH5α competent cells. The clone libraries of *amoA* and *nirS* genes were constructed for each of the 8 activated sludge samples. The clone libraries of *nirK* gene were constructed for only five samples since the other three samples failed to obtain target gene by PCR amplification. At least 100 positive clones in each library were randomly selected for sequencing. The plasmids were isolated from the positive clones using a QIAprep spin miniprep kit (Qiagen, CA, USA). The target genes were sequenced forwardly using the sequencing primer M13F on an automated ABI 3730XL DNA sequencer (Applied Biosystems) by Beijing Genomics Institute, China.

All DNA sequences obtained were aligned with those in the GenBank database of the NCBI for homology comparison. A distance matrix was calculated for all sequences, and then clustered into operational taxonomic units (OTUs) using the MOTHUR software package. To obtain the nearest phylogenetic relatives, one representative clone selected from each OTU and the references were aligned together by MEGA 6, the evolutionary distances were computed using the maximum composite likelihood method with 1000 replicates of bootstrap value. Branches corresponding to partitions reproduced in less than 50% bootstrap replicates are collapsed. The sequences of *amoA, nirK, nirS* obtained for this study have been submitted to the GenBank database under accession numbers KP732738-KP733686, KR151695-KR152194, KR073103-KR073200 and KR150995-KR151694.

### Illumina sequencing of the bacterial 16S rRNA gene

For Illumina high-throughput sequencing, the V4-V5 hypervariable regions of the bacterial 16S rRNA gene were amplified by PCR with the primer set 515F/907R (5′-GTGCCAGCMGCCGCGG-3′ and 5′-CCGTCAATTCMTTTRAGTTT-3′) using the metagenomic DNA as template. The purified PCR products were sent to the Majorbio (Shanghai, China). Each 16S rRNA V4-V5 tag was sequenced from both ends on the Illumina MiSeq platform. Quality filtering of the Illumina sequences was processed as described previously^25^. Sequences were assigned to operational taxonomic units (OTUs) with a threshold of 97% similarity using the Quantitative Insights Into Microbial Ecology (QIIME) software. A representative sequence for each OTU was aligned with the reference database SILVA. The rarefaction, bacterial community richness (Chao 1 estimator), and Shannon diversity indices (index) of each sample were generated using the QIIME software. The 16S rRNA sequences obtained for this study have been deposited into the NCBI SRA database with an accession number of PRJNA280028.

### Quantitative real-time PCR

The transcript abundances of the *amoA, nirS* and *nirK* genes were quantified in triplicate by quantitative real-time PCR (qRT-PCR) assays. The qRT-PCR was performed in 20 μl of reaction mixtures containing 10 μl of SYBR Premix ExTaq mix (TaKaRa, Dalian, China), 20 ng of cDNA template, and 0.2 μl of each primer (10 μM), using an Eppendorf qRT-PCR system (Eppendorf Realplax2, Hamburg, Germany). Thermal cycling conditions were as follows: 95 °C for 10 min, followed by 40 cycles of denaturation at 95 °C for 20 s, annealing at the given temperatures for 20 s, and extension at 72 °C for 30 s. Standard curves were obtained using serial dilutions (10^2^–10^8^ for 16S rRNA and *amoA* genes, 10^1^–10^7^ for *nirS* and *nirK* genes) of plasmid containing the target genes. The specificity of the qRT-PCR amplification was determined by the melting curve. The relative gene transcription ratio was calculated following the comparative C_T_ method, which was developed by Schmittgen and Livak^26^. The details are described in Eqs 1 and 2.


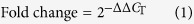



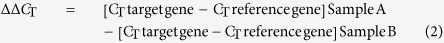


In this study, the 16S rRNA was chosen as the internal reference gene, and qRT-PCR data were expressed as the fold change of the gene transcription level.

## Additional Information

**How to cite this article**: Che, Y. *et al*. Elucidation of major contributors involved in nitrogen removal and transcription level of nitrogen-cycling genes in activated sludge from WWTPs. *Sci. Rep.* **7**, 44728; doi: 10.1038/srep44728 (2017).

**Publisher's note:** Springer Nature remains neutral with regard to jurisdictional claims in published maps and institutional affiliations.

## Supplementary Material

Supplementary Information
