# Supplementary material for: Elucidation of major contributors involved in nitrogen removal and transcription level of nitrogen-cycling genes in activated sludge from WWTPs
Source: Sci Rep. 2017 Mar 15;7:44728. doi: 10.1038/srep44728 (PMC5353630; doi:10.1038/srep44728)
Supplement: Supplementary Information [file srep44728-s1.doc]

Elucidation of major contributors involved in nitrogen removal and transcription level of nitrogen-cycling genes in activated sludge from WWTPs

You Che,^1^ Peixin Liang,^1^ Ting Gong,^1^ Xiangyu Cao,^2^ Ying Zhao,^3^ Chao Yang^1*^ Cunjiang Song,^1,4 *^

^1^Key Laboratory of Molecular Microbiology and Technology for Ministry of Education, Nankai University, Tianjin 300071, China

^2^School of Life Science, Liaoning University, Shenyang 110036, China

^3^Department of Biological Science, Jining Medical University, Jining 272067, China

^4^Department of Microbiology, College of Life Sciences, Nankai University, Tianjin 300071, China

*Corresponding authors:

Chao Yang (Tel./fax: 86 22 2350 3866; E-mail: yang_chao2008@hotmail.com)

Cunjiang Song (Tel./fax: 86 22 2350 3866; E-mail: songcj@nankai.edu.cn)

**Running title:** major contributors and key factors for nitrogen removal

**Table S1.** Effective sequences, numbers of OTUs, GOOD’s coverage, Chao1, Shannon indices of eight activated sludge samples.

| Samples | No. of effective sequences | No. of OTUs^a^ | Coverage | Chao1 | Shannon |
| --- | --- | --- | --- | --- | --- |
| L1 | 7773 | 467 | 0.98 | 557 | 4.74 |
| L2 | 7297 | 458 | 0.98 | 546 | 4.6 |
| TJ | 7151 | 447 | 0.98 | 583 | 4.43 |
| DL | 6246 | 539 | 0.97 | 625 | 4.89 |
| DZ | 8928 | 465 | 0.98 | 572 | 4.84 |
| RZ | 8850 | 526 | 0.98 | 614 | 4.52 |
| FJ1 | 6647 | 221 | 0.99 | 259 | 3.81 |
| FJ2 | 9914 | 326 | 0.99 | 384 | 4.02 |

**a.** Numbers of OTUs were calculated at the cutoff level of 0.03.

**Table S2. The relative abundance of the shared thirty genera in eight activated sludge samples^a^.**

| Genus | FJ1 | FJ2 | DZ | RZ | L1 | L2 | DL | TJ |
| --- | --- | --- | --- | --- | --- | --- | --- | --- |
| *Thauera* | 0.0107 | 0.0247 | 0.0020 | 0.0119 | 0.0012 | 0.0009 | 0.1396 | 0.0733 |
| *Planctomyces* | 0.0018 | 0.0174 | 0.0127 | 0.0118 | 0.0086 | 0.0074 | 0.0113 | 0.0052 |
| *Rubrivivax* | 0.0007 | 0.0052 | 0.0090 | 0.0117 | 0.0159 | 0.0187 | 0.0061 | 0.0022 |
| *Rhodobacter* | 0.0142 | 0.0016 | 0.0008 | 0.0049 | 0.0212 | 0.0143 | 0.0015 | 0.0043 |
| *Limnobacter* | 0.0003 | 0.0160 | 0.0058 | 0.0022 | 0.0004 | 0.0003 | 0.0099 | 0.0102 |
| *Aquabacterium* | 0.0008 | 0.0015 | 0.0006 | 0.0046 | 0.0083 | 0.0075 | 0.0074 | 0.0020 |
| *Hyphomicrobium* | 0.0020 | 0.0024 | 0.0016 | 0.0009 | 0.0082 | 0.0104 | 0.0005 | 0.0055 |
| *Thermomonas* | 0.0064 | 0.0009 | 0.0001 | 0.0013 | 0.0011 | 0.0007 | 0.0056 | 0.0112 |
| *BD1-7_clade* | 0.0008 | 0.0001 | 0.0034 | 0.0006 | 0.0029 | 0.0035 | 0.0062 | 0.0020 |
| *Paracoccus* | 0.0061 | 0.0002 | 0.0001 | 0.0001 | 0.0035 | 0.0040 | 0.0005 | 0.0043 |
| *Lautropia* | 0.0038 | 0.0002 | 0.0008 | 0.0004 | 0.0045 | 0.0035 | 0.0019 | 0.0013 |
| *Paludibacter* | 0.0040 | 0.0001 | 0.0006 | 0.0007 | 0.0003 | 0.0004 | 0.0034 | 0.0006 |
| *Macellibacteroides* | 0.0030 | 0.0003 | 0.0026 | 0.0009 | 0.0004 | 0.0006 | 0.0008 | 0.0013 |
| *Afipia* | 0.0005 | 0.0015 | 0.0009 | 0.0009 | 0.0023 | 0.0016 | 0.0002 | 0.0010 |
| *Brachymonas* | 0.0892 | 0.0081 | 0.0001 | 0.0002 | 0.0000 | 0.0004 | 0.0005 | 0.0003 |
| *Nitrospira* | 0.0000 | 0.0015 | 0.0063 | 0.0238 | 0.0017 | 0.0024 | 0.0401 | 0.0092 |
| *Dechloromonas* | 0.0000 | 0.0021 | 0.0095 | 0.0039 | 0.0298 | 0.0234 | 0.0052 | 0.0019 |
| *Thiobacillus* | 0.0000 | 0.0002 | 0.0002 | 0.0001 | 0.0012 | 0.0013 | 0.0178 | 0.0469 |
| *Terrimonas* | 0.0000 | 0.0122 | 0.0004 | 0.0018 | 0.0144 | 0.0105 | 0.0039 | 0.0197 |
| *Acinetobacter* | 0.0553 | 0.0001 | 0.0001 | 0.0004 | 0.0023 | 0.0024 | 0.0003 | 0.0000 |
| *Nitrosomonas* | 0.0000 | 0.0002 | 0.0148 | 0.0037 | 0.0122 | 0.0106 | 0.0076 | 0.0042 |
| *Candidatus_Competibacter* | 0.0000 | 0.0129 | 0.0163 | 0.0002 | 0.0016 | 0.0010 | 0.0003 | 0.0001 |
| *Sulfuritalea* | 0.0000 | 0.0004 | 0.0046 | 0.0034 | 0.0057 | 0.0070 | 0.0077 | 0.0029 |
| *Ornatilinea* | 0.0000 | 0.0003 | 0.0007 | 0.0001 | 0.0008 | 0.0026 | 0.0037 | 0.0194 |
| *Pirellula* | 0.0005 | 0.0000 | 0.0029 | 0.0062 | 0.0041 | 0.0031 | 0.0052 | 0.0035 |
| *Longilinea* | 0.0064 | 0.0023 | 0.0000 | 0.0015 | 0.0021 | 0.0017 | 0.0030 | 0.0058 |
| *Gemmata* | 0.0000 | 0.0019 | 0.0005 | 0.0022 | 0.0033 | 0.0026 | 0.0051 | 0.0020 |
| *Bryobacter* | 0.0000 | 0.0001 | 0.0035 | 0.0028 | 0.0020 | 0.0021 | 0.0035 | 0.0010 |
| *Haliangium* | 0.0000 | 0.0024 | 0.0007 | 0.0034 | 0.0016 | 0.0006 | 0.0024 | 0.0014 |
| *Blastopirellula* | 0.0003 | 0.0000 | 0.0007 | 0.0006 | 0.0009 | 0.0013 | 0.0039 | 0.0023 |

**a.** The abundance is presented in terms of the percentage of the total sequences.

**Table S3.** Description and operational parameters of the eight WWTPs.

| WWTP | City | Process | Latitude (N) | Longitude (E) | Influent (mg l^-1^) | | | Effluent (mg l^-1^) | | | pH | DO  (mg l^-1^) | Temp  (°C) | HRT  (h) | SRT  (d) | MLSS  (mg l^-1^) | RAS  (%) |
| --- | --- | --- | --- | --- | --- | --- | --- | --- | --- | --- | --- | --- | --- | --- | --- | --- | --- |
|  |  |  |  |  | COD | NH4^+^ | TN | COD | NH4^+^ | TN |  |  |  |  |  |  |  |
| L1 | Liaoning | A/O | 41°48′1.96″ | 123°25′12.02″ | 285 | 15 | 20 | 50 | 3.0 | 6.0 | 7.7 | 2.4 | 14.2 | 18 | 10-15 | 3500 | 50-100 |
| L2 | Liaoning | A/O | 41°06′37.62″ | 122°59′45.09″ | 270 | 20 | 25 | 45 | 3.5 | 7.0 | 7.5 | 2.0 | 15.1 | 20 | 8-15 | 4000 | 60-100 |
| TJ | Tianjin | A/O | 39°01′39.42″ | 117°06′48.13″ | 300 | 40 | 65 | 50 | 7.4 | 20 | 7.9 | 2.3 | 19.0 | 13 | 13-15 | 3500 | 70-100 |
| DL | Tianjin | A/O | 39°07′57.66″ | 117°24′5.80″ | 490 | 38 | 60 | 20 | 2.8 | 19 | 7.4 | 2.1 | 19.6 | 13 | 11-15 | 3700 | 50-100 |
| DZ | Shandong | A/O | 37°27′15.38″ | 116°18′56.66″ | 290 | 40 | 50 | 30 | 3.0 | 11 | 7.8 | 2.0 | 22.0 | 16 | 14-17 | 4500 | 50-100 |
| RZ | Shandong | A/O | 35°24′51.01″ | 119°29′42.74″ | 400 | 30 | 53 | 60 | 5.0 | 20 | 7.3 | 2.8 | 21.2 | 12 | 13-15 | 4000 | 75-100 |
| FJ1 | Fujian | A/O | 24°31′55.38″ | 117°54′4.65″ | 200 | 35 | 50 | 55 | 3.8 | 18 | 8.3 | 1.7 | 30.0 | 15 | 10-15 | 4300 | 50-100 |
| FJ2 | Fujian | A/O | 24°28′19.80″ | 118°10′11.29″ | 260 | 50 | 62 | 40 | 2.6 | 20 | 7.6 | 2.6 | 29.4 | 14 | 12-16 | 3400 | 50-100 |

**Table S4.** Primers used for PCR and qPCR in this study.

| Target | Primers | Sequences (5’→ 3’) | Fragment size (bp) | Annealing temp (°C) | References |
| --- | --- | --- | --- | --- | --- |
| AOB-*amoA* | amoA-1F  amoA-2R | GGGGTTTCTACTGGTGGT  CCCCTCGGCAAAGCCTTCTTC | 491 | 53 | Rotthauwe et al (1997) |
| *nirS* | nirS1F  nirS6R | CCTAYTGGCCGCCRCART  CGTTGAACTTRCCGGT | 890 | 56 | Braker et al (1998) |
|  |  |  |  |  |  |
| *nirK* | nirK1F  nirK5R | GGMATGGTKCCSTGGCA  GCCTCGATCAG(A/G)TT(A/G)TGG | 514 | 56 | Braker et al (1998) |
|  |  |  |  |  |  |
| AOB-*amoA* (qPCR) | amoA-1F | GGGGTTTCTACTGGTGGT | 491 | 58 | Rotthauwe et al (1997) |
|  | amoA-2R | CCCCTCGGCAAAGCCTTCTTC |  |  |  |
| *nirS*  (qPCR) | nirS2F | TACCACCC(C/G)GA(A/G)CCGCGCGT | 165 | 58 | Braker et al (1998) |
|  | nirS3R | GCCGCCGTC(A/G)TG(A/C/G)AGGAA |  |  |  |
| *nirK*  (qPCR) | nirK 876F | ATYGGCGGVAYGGCGA | 165 | 58 | Henry et al (2004) |
|  | nirK 1040R | GCCTCGATCAGRTTRTGGTT |  |  |  |
| 16S rRNA  (qPCR) | 341F | CCTACGGGAGGCAGCAG | 174 | 58 | López-Gutiérrez et al (2004) |
|  | 515R | ATTCCGCGGCTGGCA |  |  |  |


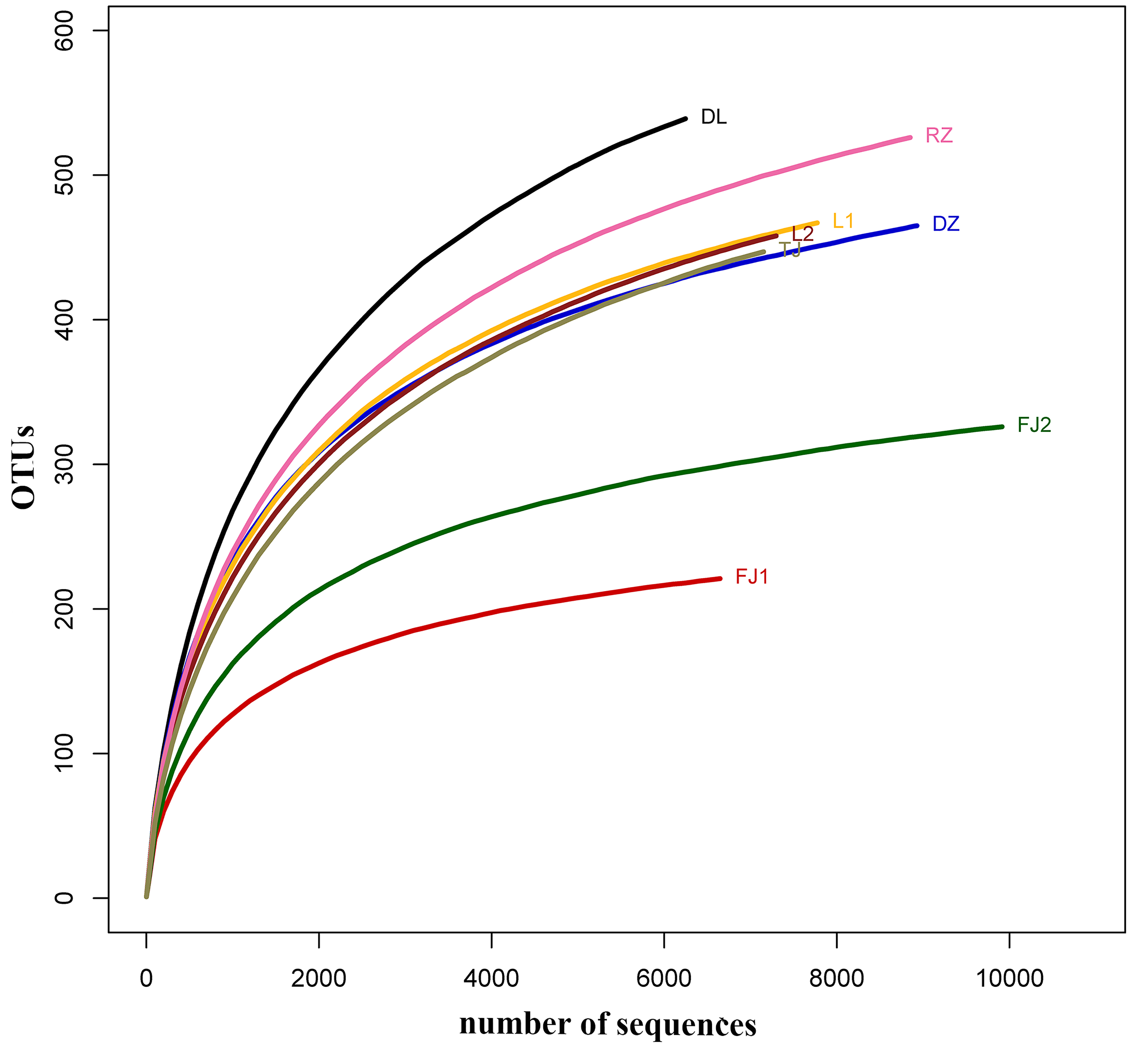


**Fig. S1.** Rarefaction curves of 8 activated sludge samples at cutoff level of 3%.


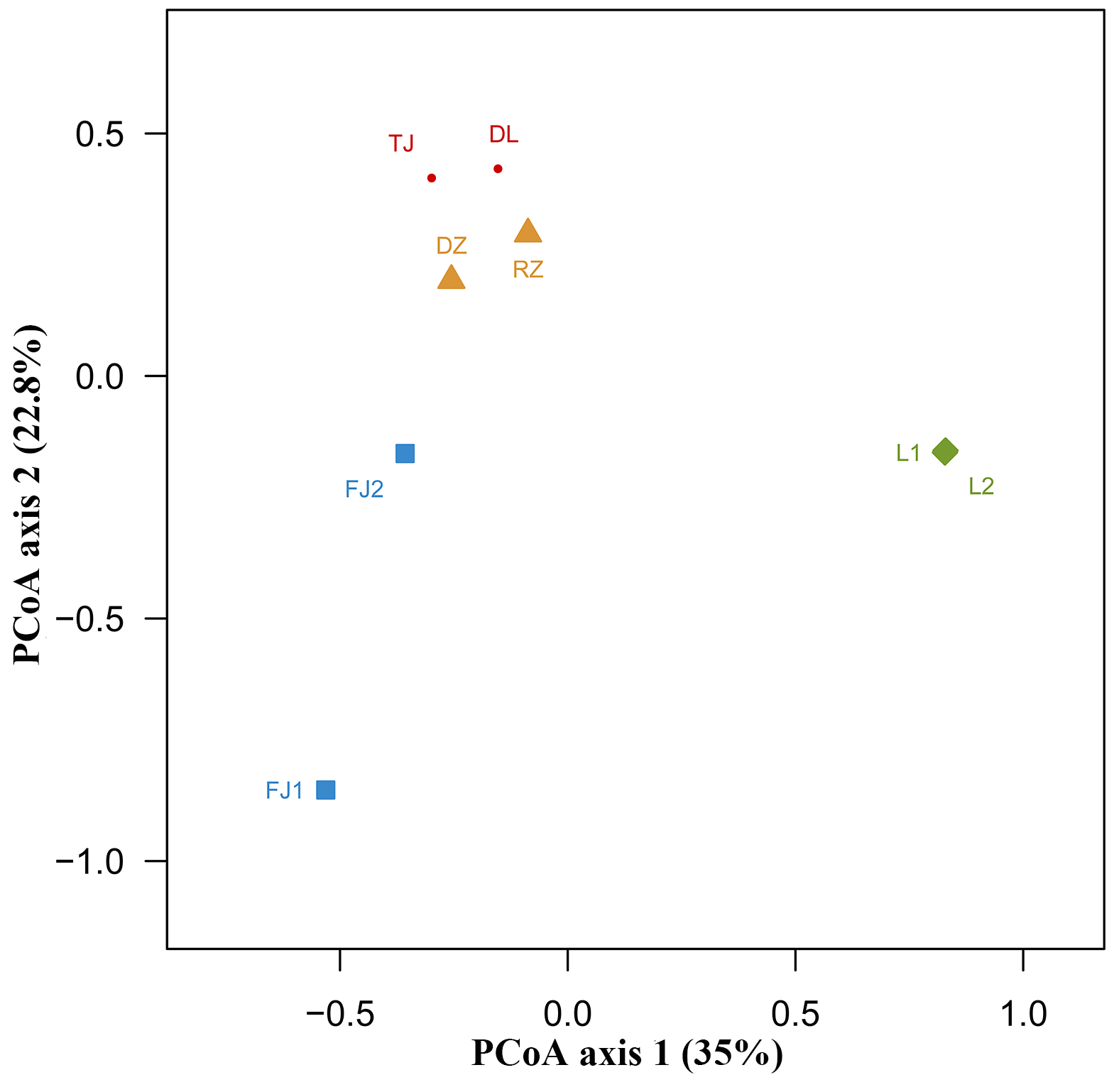


**Fig. S2.** Principal coordinates analysis (PCoA) ordinations of Bray-Curtis similarities calculated based on OTUs at cutoff level of 3% showing the bacterial community difference of the 8 activated sludge samples.


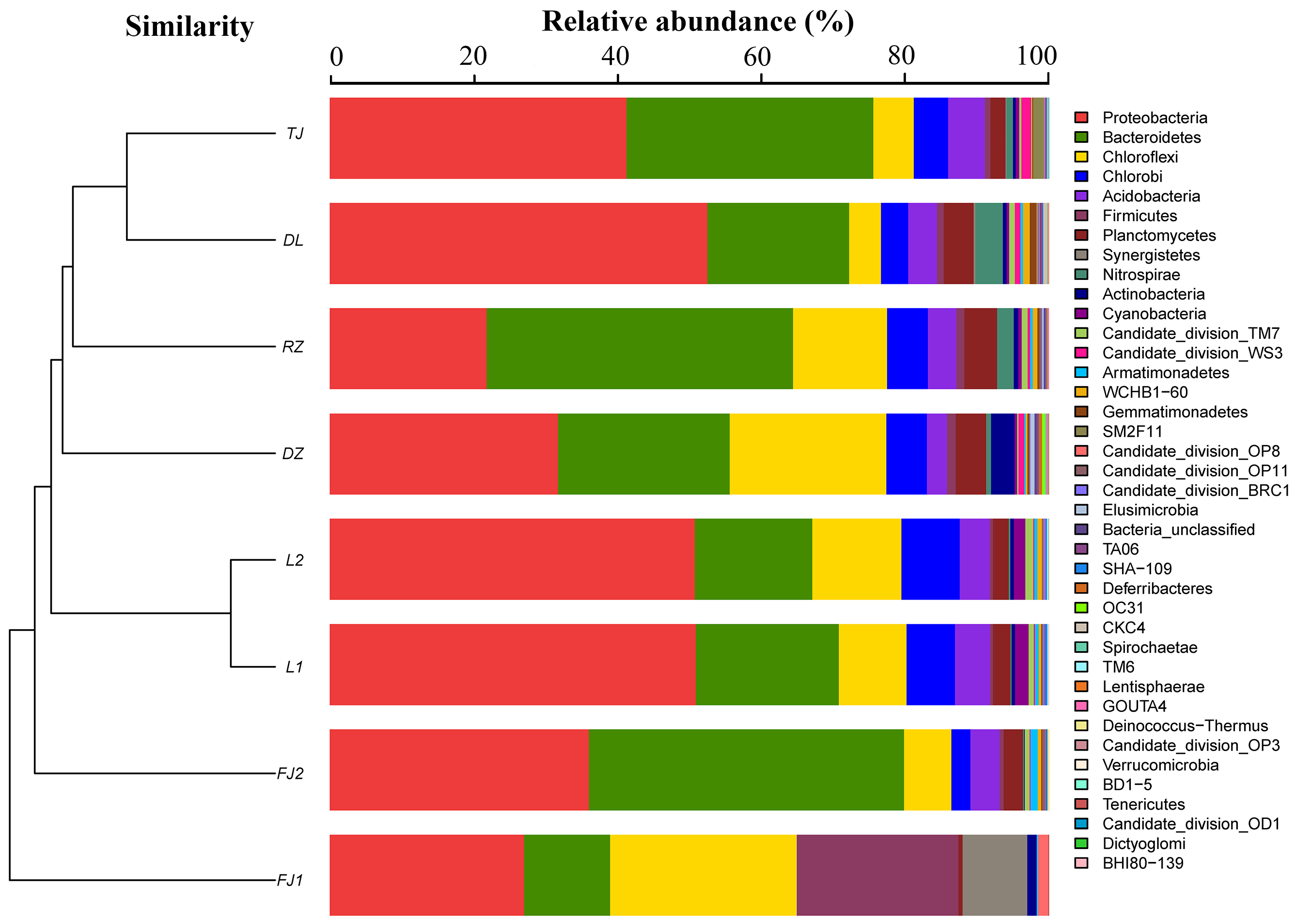


**Fig. S3.** Abundance of different phyla in the 8 activated sludge samples. UPGMA clustering used to interpret the diversity of the microbial communities based on Bray-Curtis similarity index.


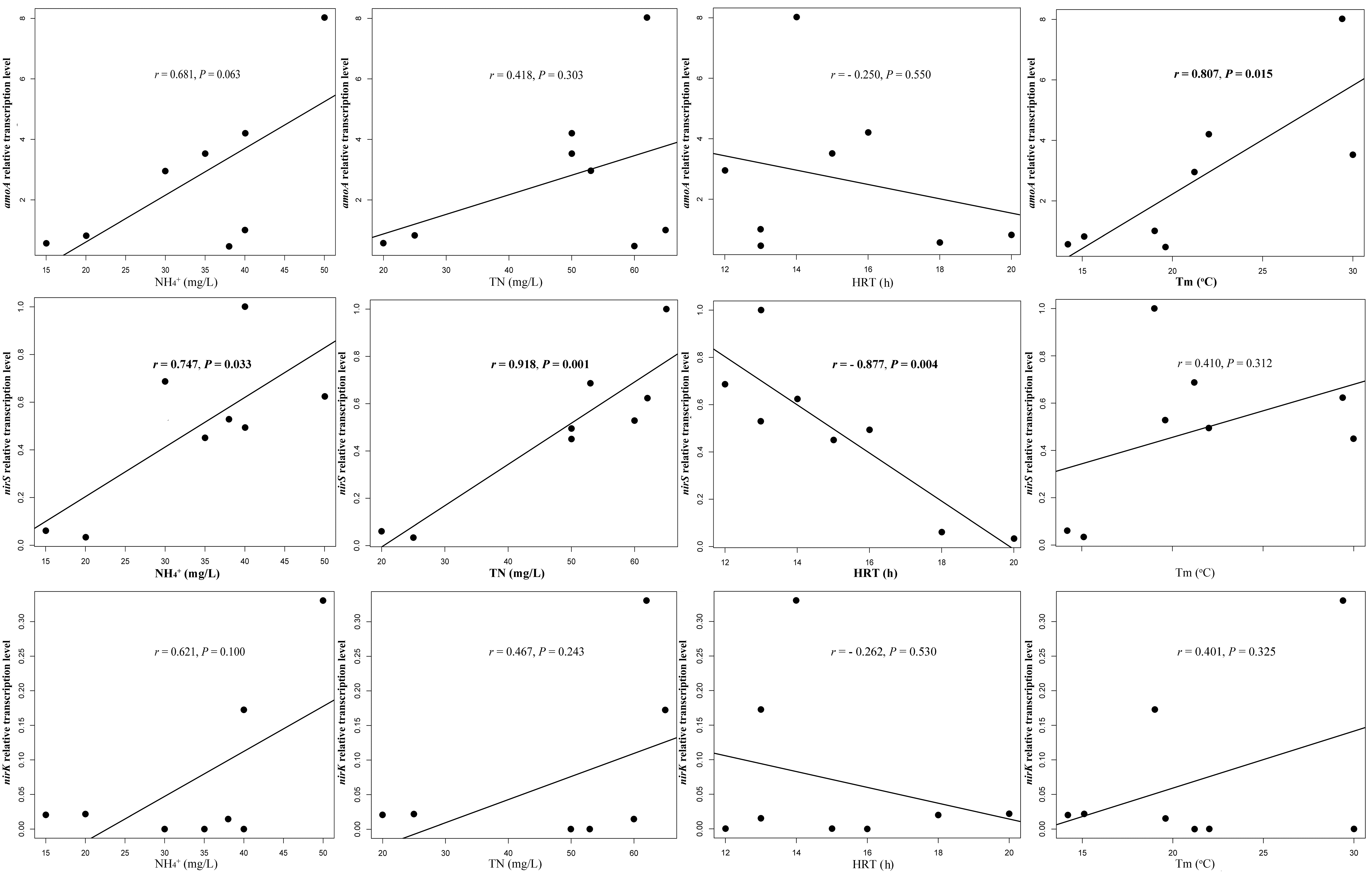


**Fig. S4.** Linear regressions were used to test the Pearson correlation between relative transcription level of functional genes (*amoA*, *nirS* and *nirK*) and different parameters including influent NH_4_^+^, TN, HRT or Tm, significant variables (*p* < 0.05) correlated to transcription level are represented as bold.

**References**

Braker, G., Fesefeldt, A. & Witzel, K.P. Development of PCR primer systems for amplification of nitrite reductase genes (*nirK* and *nirS*) to detect denitrifying bacteria in environmental samples. *Appl Environ Microbiol*. **64**, 3769–3775 (1998).

Henry, S. *et al*. Quantification of denitrifying bacteria in soils by *nirK* gene targeted real-time PCR. *J Microbiol Methods*. **59**, 327–335 (2004).

López-Gutiérrez, J.C. *et al*. Quantification of a novel group of nitrate-reducing bacteria in the environment by real-time PCR. *J Microbiol Methods*. **57**, 399–407 (2004).

Rotthauwe, J.H., Witzel, K.P. & Liesack, W. The ammonia monooxygenase structural gene *amoA* as a functional marker: molecular fine-scale analysis of natural ammonia-oxidizing populations. *Appl Environ Microbiol*. **63**, 4704–4712 (1997).
